# The Golden Ratio in Pediatric Wrist Anatomy: A Divine Symmetry

**DOI:** 10.7759/cureus.26939

**Published:** 2022-07-17

**Authors:** Georgios Mamarelis, Edward Karam, Mohammad Z Sohail, Steve Key

**Affiliations:** 1 Trauma & Orthopaedics, Royal London Hospital, London, GBR; 2 Orthopaedics, Addenbrooke's Hospital, Cambridge, GBR; 3 Orthopaedics, Royal National Orthopaedic Hospital, Stanmore, GBR

**Keywords:** anatomy at the wrist, articular anatomy, body symmetry, distal end radius, golden ratio

## Abstract

Introduction: The golden ratio, which equals 1.61803…, and is usually defined by the Greek letter φ (phi), has attracted broad attention for a long time. It has been found in many phenomena in the universe including, body symmetry and locomotion. Within this context, the purpose of our study was to evaluate normal morphometric measurements of the wrist in the pediatric population and to identify if phi (φ) is part of the distal radioulnar joint.

Methods: We retrospectively reviewed the hospital records of all skeletally immature patients requiring surgical intervention for distal radius fracture in our unit between January 2010 and January 2017. We define and describe a reproducible method to measure the ratio of the distal radial and ulnar physes.

Results: A total of 268 patients were included with a mean age of 9.41 (3-16) years and a mode of 7 years. Some 63.4% were boys -- 43.3% were right-sided injuries and 56.7% were left-sided injuries. The ratio between the total width of the radial and ulnar growth plates and the radial growth plate closely approximated φ; the mean of this ratio in all the patients included was 1.619684 (1.5848-1.6643). Most of the injuries happened in the summertime, between May and August.

Conclusion: We found that the golden ratio exists in our body to play its harmony in the pediatric wrist joint. We believe that with the support of further studies, the golden ratio might yield diagnostic and prognostic implications in the treatment of distal radius/ulnar fractures or abnormalities in this population.

## Introduction

The golden ratio commonly appears in mathematics, architecture, art, and other areas, and equates approximately to 1.618 [1]. In mathematics, the golden ratio is calculated by dividing a segment in two, such that the proportion between the segment and its longer part will be equal to the proportion between the longer and shorter part [2].

The first known written definition can be found in Euclid’s Elements book [3]. At the beginning of the 12th century, the mathematician Mark Barr introduced the symbol phi (φ) for the golden ratio. It was the first letter of the name Phidias, a famous 5th century BC Greek sculptor, architect, and painter who used the golden ratio to form the basis of his sculptures [1]. 

Since ancient Greek times, it is believed that the golden ratio can be found in nature and human body anthropometry, despite the paucity of scientific literature on the topic. In our study, we focus on the association of the number φ with the pediatric distal radial and ulnar anatomy. Distal radius fractures are common within the pediatric population, accounting for up to 20%-30% of all injuries [4-5]. The distal radial metaphysis is the most common site of these fractures [4].

According to AO (Arbeitsgemeinschaft für Osteosynthesefragen) pediatric comprehensive classification of long bone fractures, we can identify the metaphysis by a square whose side is the same length as the widest part of the growth plate. In the case of bone pairs such as radius/ulna and tibia/fibula, then both bones should be included in the square [6].

Our hypothesis was that the ratio between the total width of the radial and ulnar growth plates to the radial growth plate will be the same as the ratio of the radial growth plate to the ulnar growth plate and should, therefore, approximate the golden ratio.

## Materials and methods

Methods

**Figure 1:**
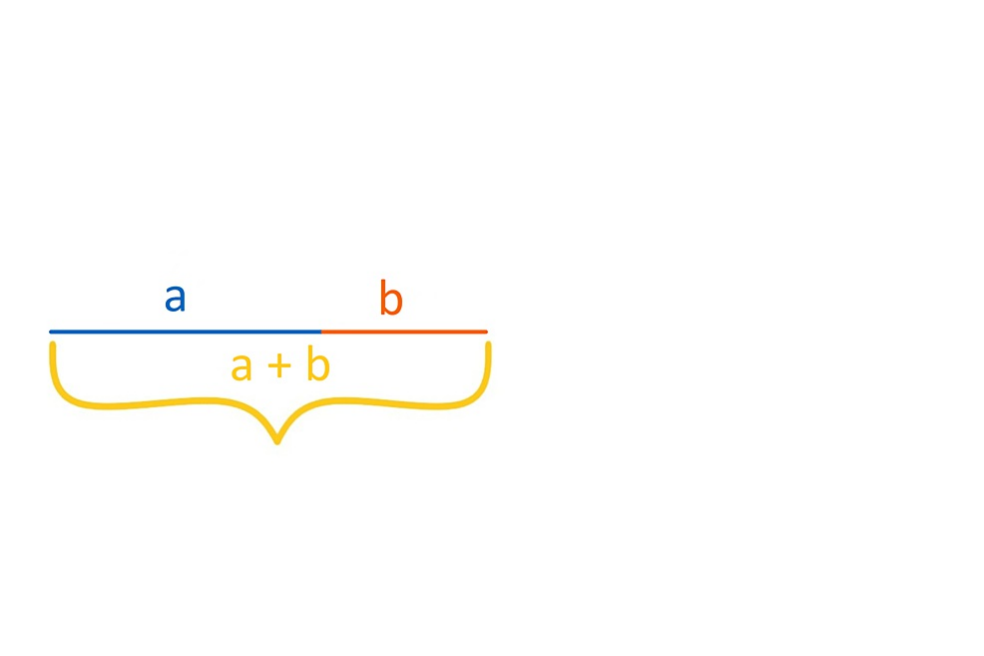
Golden ratio – line segments.

**Figure 2:**
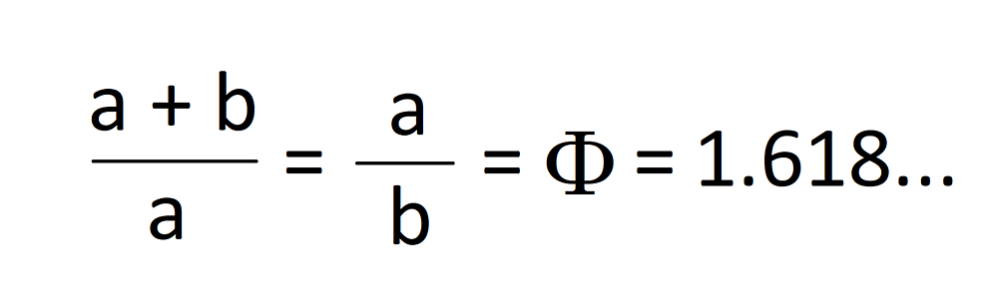
Golden ratio proportion -- algebraic form.

**Figure 3:**
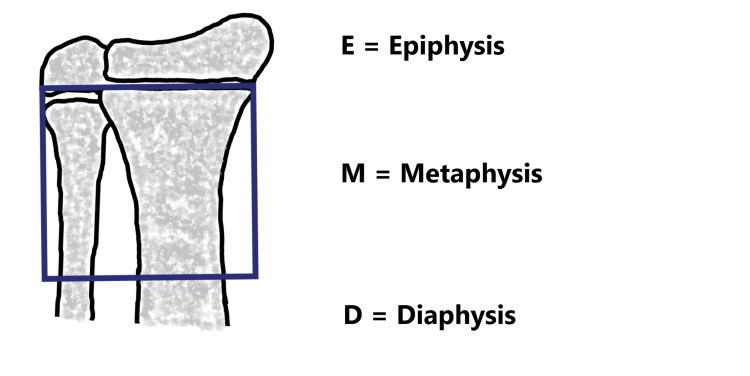
Diagrammatic measurement of metaphysis.

In this retrospective observational study, we included all skeletally immature patients assessed radiologically, who sustained a distal radius fracture and underwent a procedure in operating theaters between January 2010 and July 2017. Patients with partially or fully closed growth plates were excluded (Figures 1-3).

Distal radius and ulnar physis calculation

**Figure 4:**
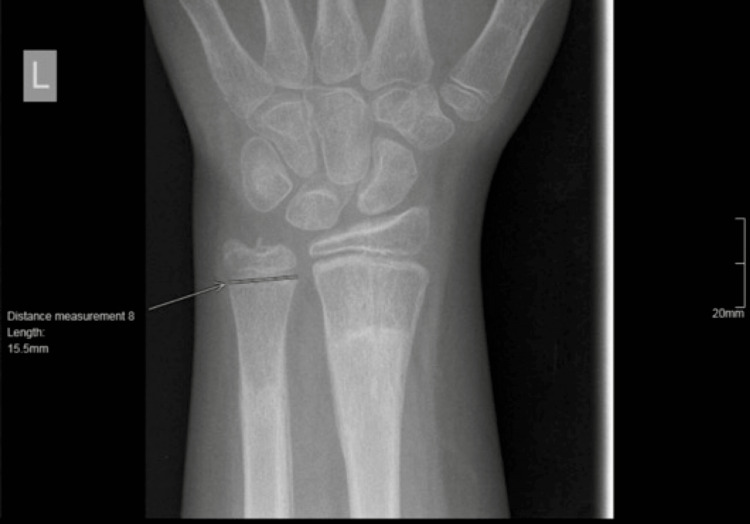
Measurement of the widest dimension of the ulnar physis.

**Figure 5:**
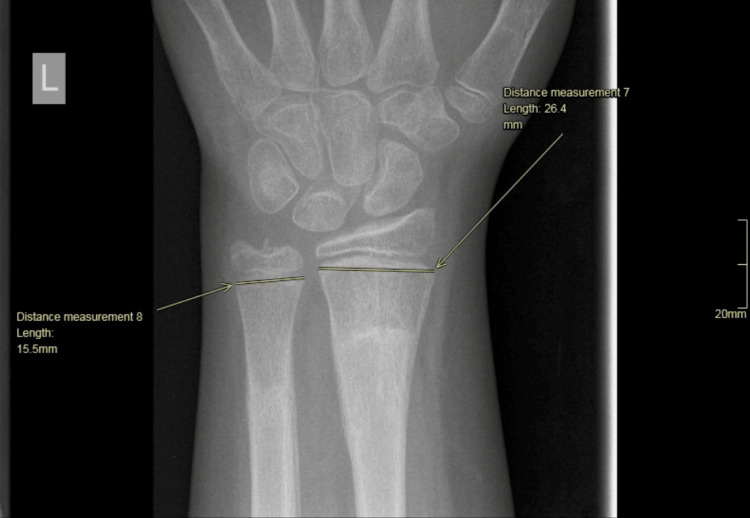
Measurement of the widest dimension of the radial physis.

We developed a reproducible method to measure the ratio of the distal radial and ulnar physes. All measurements were done in a true posteroanterior (PA) radiograph of the distal radius. First, we measured the widest mediolateral dimension of the ulnar physis (Figure 4). Subsequently, we measured the widest mediolateral dimension of the radial physis (Figure 5). 

**Figure 6:**
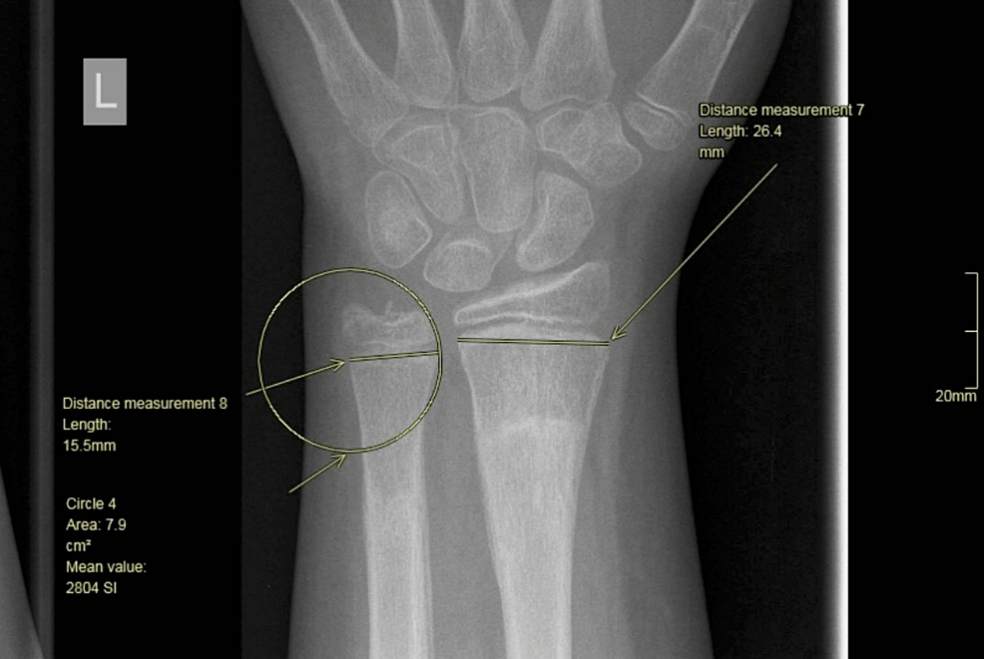
Drawing of a circle centered on the ulnar side of the ulnar physis (circle radius is the width of the ulnar physis).

We then drew a circle centered on the ulnar side of the ulnar physis, the circle’s radius being the ulnar physis (Figure 6).

**Figure 7:**
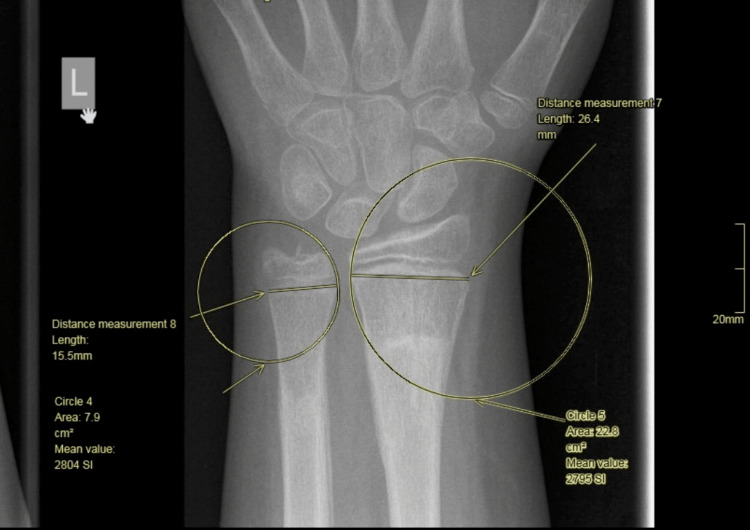
Drawing of a circle centered on the radial aspect of the radius physis (circle radius is the width of the radial physis).

We then drew a circle centred over the radial aspect of the radial physis and with the radius of the circle equal to the radial physeal length (Figure 7). 

**Figure 8:**
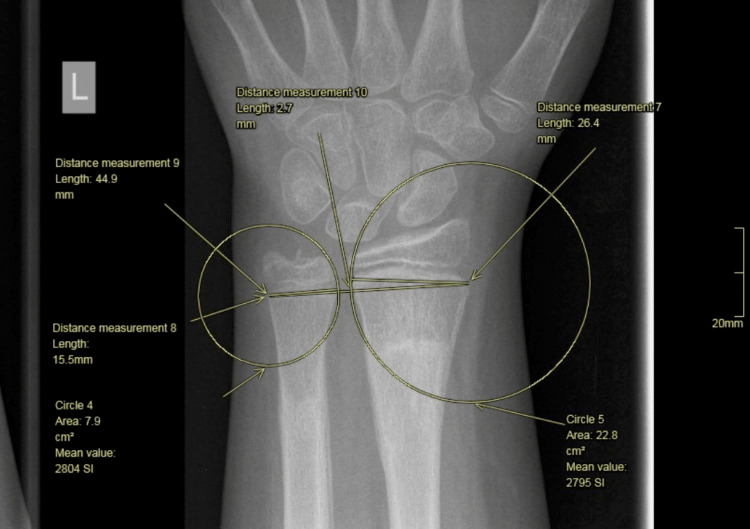
Line drawn between the centers of the two circles.

**Figure 9:**
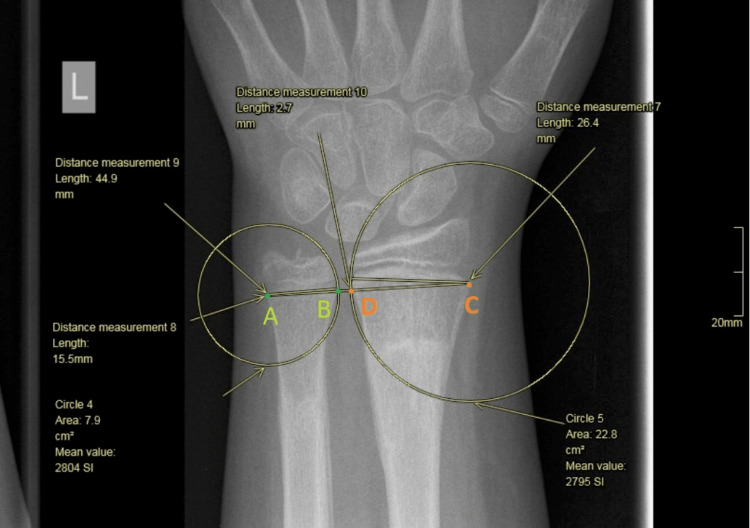
AB distance corresponds to the ulnar physis. DC corresponds to the physis of the radius. BD corresponds to the radioulnar joint.

Then we drew a line between the two centers of these circles and we measured the part of the line which was outside of the circles. We divided this line by 2, as this is the space of the distal radioulnar joint that participated equally into the wrist joint. Following these drawings, we calculated the proportion with the equation $\frac{CD + BD/2}{AB + BD/2}$ (Figures 8-9).

## Results

Some 268 patients were included in this study -- 63.4% were boys and 36.6% were girls. Ages ranged from 3 to 16 years old with a mean of 9.41, a median of 9, and a standard deviation of 3.246. Some 116 (43.3%) fractures were on the right side and 152 (56.7%) were on the left. No bilateral injuries were observed.

When we used our equation $\frac{CD + BD/2}{AB + BD/2}$ to calculate the proportion of the radial and ulna physis, we found that the result is approximately equal to the number φ. The mean of all our patients was 1.619684 (1.5848-1.6643) and the standard deviation was 0.0179473. The frequency of each age group with an average of phi is shown in Table 1. 

**Table 1:** Average measurement of our equation in relationship to age.

Age	Frequency	Average measurements
3	5	1.618912731
4	8	1.615254249
5	20	1.621134699
6	26	1.617770116
7	34	1.621147302
8	24	1.620943379
9	18	1.611171123
10	26	1.623010678
11	24	1.629361125
12	29	1.621080288
13	17	1.616227367
14	25	1.615250012
15	8	1.610687803
16	4	1.626401356
Total	268	1.619168016

## Discussion

The golden ratio φ is a proportion that has been found with many natural phenomena, as well as in different fields in medicine: cardiovascular pathophysiology [7-9], respiratory medicine [10], ENT surgery [11], dental surgery [12-13], plastic surgery [14-16], and many other fields [17-18]. To the best of our knowledge, this is the first study to assess the geometry of the wrist joint in relation to the golden ratio.

In our study, we determined that the golden ratio is part of the anatomy of the wrist joint. Our measurements showed that the ratio between the total width of radial and ulnar growth plates to radial growth plate was almost equal with the number φ= 1.61803. 

We believe that this observation of wrist joint anatomy could be an important factor to guide the management of distal radial fractures, as this topic remains controversial. Until now, there are no objective criteria to assess when to perform manipulation under anesthesia (MUA) or Kirschner wires (K-wires) fixation for a distal radius fracture [19-20]. The number φ as a guide for the normal radio-ulnar ratio could be an additional radiological tool in the arsenal of the orthopedic surgeon deciding treatment strategies for these injuries.

More specifically, it is still debated which deformities of the distal radial metaphysis can be accepted for either MUA or fixation with K-wire [21-22]. There are few studies that define a diametaphyseal transitional zone. These characterize the diametaphyseal transitional zone as the area which remains when the square over the radial physis has been subtracted from the metaphysis [23-24]. This definition can be a useful factor to determine the required surgical technique [23, 25]. The radio-ulnar ratio described in our study is similar in concept to the transitional zone and we believe its clinical relevance in management decision-making can be linked to the findings of transitional zone studies. However, this specific point is outside of the scope of this anatomical study and would require further research, also needed to investigate the mathematical regularity which can be found in the human body, as we feel that the ratio φ can be a useful guide in deciding the appropriate surgical technique for distal radius fractures.

Limitations

We acknowledge that our study has some limitations. We only included the pediatric population that had sustained distal radius fracture. Nonetheless, the measurements were completed when the normal anatomy was restored, therefore, our findings can be applied to the general pediatric population of skeletally immature patients with open physes (typically up to 14 years old for girls and 16 years old for boys).

## Conclusions

In conclusion, the golden ratio resides in our body to play its harmony in the wrist joint. Understanding the role of the golden ratio or proportion might yield diagnostic and prognostic implications in the treatment of distal radius/ulnar fractures or abnormalities.
